# Physical Activity on Prescription (PAP), in patients with metabolic risk factors. A 6-month follow-up study in primary health care

**DOI:** 10.1371/journal.pone.0175190

**Published:** 2017-04-12

**Authors:** Stefan Lundqvist, Mats Börjesson, Maria E. H. Larsson, Lars Hagberg, Åsa Cider

**Affiliations:** 1 Department of Health and Rehabilitation, Unit of Physiotherapy, Institute of Neuroscience and Physiology, Sahlgrenska Academy, University of Gothenburg, Gothenburg, Sweden; 2 Närhälsan Rehabilitation FaR-teamet central and western Gothenburg, Region Västra Götaland, Gothenburg, Sweden; 3 Department of Food and Nutrition and Sport Science, Faculty of Education, University of Gothenburg, Gothenburg, Sweden; 4 Institute of Neuroscience and Physiology, Sahlgrenska Academy, Sahlgrenska University Hospital/Östra, Gothenburg, Sweden; 5 Närhälsan Research and Development Primary Health Care, Region Västra Götaland, Gothenburg, Sweden; 6 University Health Care Research Center, Faculty of Medicine and Health, Örebro University, Örebro, Sweden; University of Oslo, NORWAY

## Abstract

There is strong evidence that inadequate physical activity (PA) leads to an increased risk of lifestyle-related diseases and premature mortality. Physical activity on prescription (PAP) is a method to increase the level of PA of patients in primary care, but needs further evaluation. The aim of this observational study was to explore the association between PAP-treatment and the PA level of patients with metabolic risk factors and the relationship between changes in the PA level and health outcomes at the 6 month follow-up. This study included 444 patients in primary care, aged 27–85 years (56% females), who were physically inactive with at least one component of metabolic syndrome. The PAP-treatment model included: individualized dialogue concerning PA, prescribed PA, and a structured follow-up. A total of 368 patients (83%) completed the 6 months of follow-up. Of these patients, 73% increased their PA level and 42% moved from an inadequate PA level to sufficient, according to public health recommendations. There were significant improvements *(p*≤ 0.05) in the following metabolic risk factors: body mass index, waist circumference, systolic blood pressure, fasting plasma glucose, cholesterol, and low density lipoprotein. There were also significant improvements regarding health-related quality of life, assessed by the Short Form 36, in: general health, vitality, social function, mental health, role limitation-physical/emotional, mental component summary, and physical component summary. Regression analysis showed a significant association between changes in the PA level and health outcomes. During the first 6-month period, the caregiver provided PAP support 1–2 times. This study indicates that an individual-based model of PAP-treatment has the potential to change people’s PA behavior with improved metabolic risk factors and self-reported quality of life at the 6 month follow-up. Thus, PAP seems to be feasible in a clinical primary care practice, with minimum effort from healthcare professionals.

## Introduction

There is strong evidence that insufficient physical activity (PA) is associated with increased risk of developing lifestyle-related diseases and premature death [1]. Metabolic syndrome (MetS) is not consistently defined, but includes: overweight, abdominal obesity, insulin resistance, dyslipidemia, and hypertension in various combinations [2]. The presence of MetS carries a high risk for developing cardiovascular disease and type 2 diabetes [3]. Importantly, MetS is also associated with physical inactivity, further aggravating the risk of cardiovascular events [4].

The definition of PA is “any bodily movement produced by skeletal muscles that results in energy expenditure” and can be categorized as e.g. a household, occupational, leisure time, and sporting activity [5]. Exercise is PA with the objective to improve or maintain physical fitness components and is categorized in terms of the type, frequency, duration, intensity, and purpose [6]. The internationally recommended minimum level of PA [7] is moderate-intensity aerobic PA 150 min per week or, alternatively, vigorous-intensity aerobic PA 75 min per week, which has been associated with a clinically relevant risk reduction. Additional health benefits can be achieved by increased PA, above the national recommendation levels [8].

Despite the evidence-based positive effects of regular PA on health, implementing PA as an integrated method of treatment in health care remains a major challenge [9]. The Swedish National Board of Health’s guidelines for disease prevention methods recommend the use of individual-based dialogue, written information, training diaries, a pedometer, and structured follow-up when the patient’s PA level is insufficient [10]. An example of such a treatment strategy is physical activity on prescription (PAP), which is individually tailored for each patient and prescribed for preventive and therapeutic purposes as a first-line treatment.

Meta-analyses of international PAP studies show varying results, with small to medium positive intervention effects when comparing increased PA levels with usual care. However, there is uncertainty due to the lack of high quality studies and further research is needed with more homogenized, comparable PAP interventions, longer follow-up, and objective measures of outcome [11, 12]. Swedish lifestyle interventions, including PAP, has shown to be cost-effective [13, 14] and the Swedish PAP intervention method had positive effects on PA levels, body composition, cardio metabolic risk factors, and health related quality of life (HRQOL) [15, 16]. Although scientific evidence has resulted in clinical treatment guidelines [10] and there are some evaluated Swedish PAP studies [15–18], PA is still underutilized as a treatment strategy in Swedish health care [19, 20]. There is still a lack of knowledge about PAP interventions suitable for different patient groups to improve their PA level and health outcomes. Further studies are needed evaluating clinical feasible PAP strategies on a large sample [21–23].

In primary care in the city of Gothenburg, health care centers have implemented PAP-treatment, individualized for patients with metabolic risk factors, with the purpose of increasing the PA level and health benefits. This specific model of PAP-treatment in daily clinical work has not been evaluated and may add new insights on how the extent of the intervention affects the PA level and health status.

The aim of this observational study was to explore the association between PAP-treatment and the PA level of patients with metabolic risk factors and the relationship between changes in the PA level and health outcomes, including metabolic risk factors and HRQOL at the 6-month follow-up.

## Methods

### Study design

This is a prospective, longitudinal observational study with a 6-month follow-up of PAP-treatment in a daily clinical primary care practice. The present study is part of an ongoing study with a 5 year follow-up.

### Study population

The study population included 444 patients, aged 27–85 years. The patients were selected as a convenience sample from 15 primary health care centers in Gothenburg center/west. The patients agreed with their health care provider to participate in the study before they were prescribed PA and were included prospectively from 2010 to 2014. The population of central/western Gothenburg is 220 000 and has a higher socio-economic status compared with Gothenburg overall [24]. The inclusion criteria were: physically inactive, having at least one component of MetS present, and receiving PAP-treatment. The patients also had to understand the Swedish language to fill in the questionnaires.

### Intervention

The patient was informed of the possibility to receive treatment with PAP by written information in the waiting room and orally by their caregiver. All authorized personnel were educated on the effects of PA according to the *Physical activity in the prevention and treatment of disease* (FYSS) [25] and the concept of the Swedish PAP model. Authorized personnel, mainly nurses, at the health care centers prescribed PA to the patients. The PAP included a dialogue with the patient, based on the principles of motivational interviewing (MI) [26]. Each patient’s previous and current level of PA and their preferences for different kinds of PAs were elucidated. Furthermore, the patient’s motivation, self-efficacy, and readiness to change PA behaviour were evaluated. This information served as the basis for the selection of the type and volume of the PA. The volume of the chosen PA was determined using the FYSS reference book, and the most suitable activity was prescribed at the appropriate relative intensity using the Borg’s rate of perceived exertion scale [27] as well as duration and frequency. To help the patient to choose a suitable PA, a registry of the local supply of PA’s was presented. It was possible to recommend two different types of PA in the PAP. This resulted, for each patient, in an individually tailored PA recommendation planned in dialogue with the patient and followed by a structured follow-up. The patients were offered individually adjusted support during the 6 month intervention period, either by revisits or telephone contacts.

### Measurements

The measurements described below were conducted when PA was first prescribed as well as at the 6-month follow-up.

#### PA level

The PA level was the primary outcome and four questionnaires were used due to the known complexity of PA assessments. 1. Self-assessment was according to the American College of Sports Medicine (ACSM) and American Heart Association (AHA) public health recommendations. The patient responded to two PA questions (ACSM/AHA questionnaire), where 30 min of moderate-intensity PA per day resulted in 1 point and 20 min of more vigorous-intensity PA per day resulted in 1.7 point during each specific day of the week. A value of <5 points indicated an inadequate PA level [28]. 2. The International Physical Activity Questionnaire (IPAQ) assessed the level of PA during the last 7 days. This instrument is extensively tested and translated into Swedish and can assess vigorous- and moderate-intensity PA, walking, and sitting time [29, 30]. 3. The Saltin-Grimby Physical Activity Level Scale (SGPALS) assessed leisure time PA during the past year at four different levels, from sedentary/physically inactive to vigorous physically active [31]. The levels have been validated against e.g. metabolic risk factors [32, 33] and the SGPALS has been published in an updated Swedish form [34]. 4. A six-grade PA scale, which is a further development of the SGPALS (Frändin/Grimby), was used and includes household activities [35]. This scale correlates with physical performance and self-assessed fitness and is used to classify PA among the elderly [36].

#### Anthropometrics

Body weight was measured with light clothing and without shoes to the nearest 0.1 kg using an electric scale (Carl Lidén AFW D300, Jönköping, Sweden). Body height was measured in an upright position without shoes to the nearest 0.5 cm using a scale fixed to the wall (Personmått PEM 136, Hultafors, Sweden), and the body mass index (BMI) was calculated. Waist circumference (WC), to the nearest 0.5 cm, was measured in a standing exhaled position, with a measuring-tape (Kirchner Wilhelm, Aspberg, Germany) placed on the patient’s skin between the lower rib and the iliac crest.

#### Systolic and Diastolic Blood Pressure (SBP, DBP)

The SBP and DBP were measured in mmHg according to guidelines [37] after 5 min rest with the patient seated with a blood pressure sphygmomanometer (Omron HEM-907, Kyoto, Japan) attached to the right upper arm at the level of the heart.

#### Blood samples

Blood samples were used to measure (in mmol/l) fasting plasma glucose (FPG) after an overnight fast, triglycerides (TG), cholesterol (Chol), High Density Lipoprotein (HDL), and Low Density Lipoprotein (LDL). Values were analyzed according to the European Accreditation system [38].

#### The cut-off values of MetS components

Cut-off values were according to the National Cholesterol Education Program (NCEP) classification and were: WC >88 cm for women, >102 cm for men; BP ≥130/85 mm Hg; FPG ≥6.1 mmol/l; TG ≥1.7 mmol/l; or HDL <1.3 mmol/l for women, <1.0 mmol/l for men [39].

#### Health related quality of life

The HRQOL was assessed with the Swedish version of the Short Form 36 (SF-36 Standard Swedish Version 1.0), which includes 36 questions [40]. It generates eight health concepts: physical functioning (PF), role physical functioning (RP), bodily pain (BP), general health (GH), vitality (VT), social function (SF), role emotional functioning (RE), and mental health (MH). The health concepts were converted to 0–100 points, where higher values represented a better HRQOL. The different health concepts of the SF-36 were also grouped into a physical component summary (PCS) and mental component summary (MCS). The SF-36 has shown good to excellent internal consistency and reliability and was validated in a representative sample of the Swedish population [40].

#### Readiness to change the PA level

The readiness to change the PA level was measured at baseline using three questions estimated on a 100 mm visual analogue scale (VAS): How prepared are you? How important is it to you? How confident are you to succeed (self-efficacy)? The questions were derived from MI and behaviour change counseling, according to Rollninck et al. [41, 42], where higher values on the VAS indicated increased readiness to change.

#### Support from the PAP-responsible nurse

The support from the nurse responsible for the PAP was assessed at the 6-month follow-up by questioning the patient about the frequency of visits at the health care center.

## Statistical analysis

Interval and ratio data are presented as the mean (m) and the dispersion as a standard deviation (SD) or 95% confidence interval (CI). Nominal and ordinal data were presented as the median (md) and minimum—maximum (min—max). A per-protocol analysis was used and differences between baseline and the 6-month follow-up, within the group, were analyzed using the paired sample t-test or Wilcoxon sign-rank test, based on the data level. Subgroup analyses between women *vs*. men and the completed group *vs*. dropout group were performed using the independent sample t-test or Mann Whitney U-test. A standardized measure of effect size (*d*) in the within-subject comparisons ($Cohen^{\prime }s\;\;d_{z}\times \sqrt{2}$) was reported to quantify the degree of differentiation in values between baseline and the 6-month follow-up. The effect size was considered small when *d* = 0.2–0.3, as medium when *d* = 0.5, and as large when *d* = 0.8 [43].

In regression analysis, both multivariate and univariate methods were used to evaluate associations between changes in the PA level and changes in health outcomes, when adjusting for potential confounders. The predictor of interest was change in PA level, calculated as a Delta (Δ)-value (6-month value minus baseline value), and the PA level at baseline, age, sex, social situation, economy, education, and smoking were examined as potential confounders. The outcomes could be classified into two clusters. The first cluster contained Δ-values of the metabolic risk factors (BMI, WC, SBP, DBP, FPG, TG, Chol, HDL, LDL) and the second cluster contained Δ-values of self-reported health, using the SF-36 HRQOL (PF, RP, BP, GH, VT, SF, RE, MH, PCS, MCS).

Univariate multiple linear regression was used to check whether or not a change in PA was significantly correlated with the nineteen independent variables, one at a time, when all the potential confounders were considered.

Multivariate linear regression was then used to test if changes in PA were significantly associated with the two clusters (change in metabolic risk factors and change in self-rated health) and not just the specific variables in the clusters. The significance was tested using a regression-based MANOVA and test-statistic for Pillai’s trace. Assumptions of normality, linearity, and outliers were checked using residual plots. All data in regression analysis were analyzed using SPSS for Windows, version 23 (IBM Corp. Armonk, NY, USA). A *p*-value <0.05 was considered statistically significant.

The patient’s contact frequency with the PAP-responsible nurse was categorized in 1–2, 3–5, 6–10, 11–20, and ≥21 contacts. All statistical analyses, except the regression analysis, were calculated in SPSS version 22.0 (IBM Corp. in Armonk, NY, USA). Statistical significance was set at *p* ≤0.05.

## Ethical aspects

The study was approved by the Regional Ethical Review Board in Gothenburg, Sweden (Dnr 678–14).

## Results

### Study population

**Fig 1:**
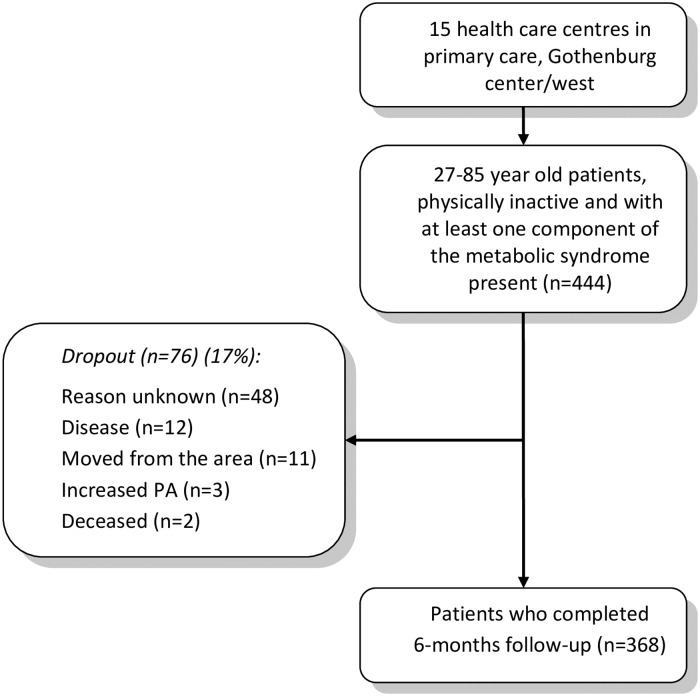
Flow of patients involved in the study. The patients were recruited from 15 health care centers.

Of the 444 included individuals, 368 completed the 6 months of follow-up. The dropout rate was 17% (Fig 1).

### Baseline characteristics

The mean age of the study population was 57 years, with 56% female. Overweight/obesity was present in 91%, hypertension in 78%, and 58% had hyperlipidemia (Table 1). Two components of MetS, WC (>88 cm for women, >102 cm for men) and BP (≥130/85 mm Hg) were present in 72% and WC (>88 cm for women, >102 cm for men) and TG (≥1.7 mmol/l) in 53%. At baseline, 61% were taking medications for components of MetS, including 54% for hypertension and 22% for hyperlipidemia. The PA level was estimated to be low, using all four PA instruments. A total of 36% of subjects were sedentary according to SGPALS, 80% reported PA equivalent to a 30 min brisk walk three times per week or less and 47% reported ≤1 time per week, according to the ACSM/AHA questionnaire (Table 2).

**Table 1:** Baseline characteristics of the follow-up and dropout group.

Variable^a^	Follow-up (n = 368)	Dropout (n = 76)	*p* value^b^
**Age**—years	57.4 (10.9)	57.6 (13.1)	0.955
**Sex**			**0.011**
Female	198 (53.8)	53 (69.7)	
Male	170 (46.2)	23 (30.3)	
**Nationality**			0.915
Sweden	312 (86.0)	62 (84.9)	
Other	51 (14.0)	11 (15.1)	
**Social situation**			0.144
Single	135 (37.9)	35 (48.6)	
Married/ cohabit	205 (57.6)	33 (45.8)	
Other	16 (4.5)	4 (5.6)	
**Economy**—perceived			0.467
Good	213 (59.3)	36 (50.7)	
Neither nor	107 (29.8)	19 (26.8)	
Bad	39 (10.9)	16 (22.5)	
**Education**			0.117
Elementary grade	69 (19.2)	14 (19.4)	
Upper secondary school	131 (36.4)	36 (50)	
University college	160 (44.4)	22 (30.6)	
**Tobacco**			0.871
Smokers	34 (9.5)	10 (13.9)	
Non-smokers	229 (63.8)	41 (56.9)	
Ex-smokers	96 (26.7)	21 (29.2)	
**Part of metabolic syndrome**			
Overweight/Obesity	333 (90.5)	71 (93.4)	0.245
Hyperglycemia	144 (39.1)	30 (39.5)	0.672
Hypertension	293 (79.6)	53 (69.7)	0.117
Hyperlipidemia	212 (57.6)	41 (53.9)	0.801
Other diagnosis			
Mental health, depression	52 (14.1)	13 (17.1)	0.446
Musculoskeletal disorders	58 (15.8)	19 (25)	**0.040**
Other	155 (42.1)	38 (50)	0.172
**Drug treatment**			
Overweight/Obesity	1 (0.3)	1 (1.3)	0.207
Hyperglycemia	46 (12.5)	13 (17.1)	0.246
Hypertension	196 (53.3)	40 (52.6)	0.901
Hyperlipidemia	77 (20.9)	17 (22.4)	0.694
Other drug treatment			
Mental health, depression	52 (14.1)	12 (15.8)	0.642
Musculoskeletal disorders	49 (13.3)	11 (14.5)	0.723
Other	132 (35.9)	35 (46.1)	0.064

^a^ Age data are given as mean (standard deviation) and data for other variables are given as number (percentage).

^b^ Difference between follow-up and dropout group. *P*-value for age was determined by an independent samples *t*-test and all the other characteristics were determined by Mann-Whitney U-test.

Statistical significance was set at *p*≤ 0.05.

**Table 2:** Baseline characteristics in anthropometrics, metabolic risk factors, physical activity level and health related quality of life—Follow-up and dropout group.

Variable^a^	Follow-up (n = 368)	Dropout (n = 76)	*p* value^b^
BMI, kg/m^2^	32.0 (5.2)	33.0 (5.8)	0.104
Waist circumference, cm	107.9 (13.1)	109.2 (13.5)	0.423
Blood pressure, mm/Hg:			
Systolic	137.3 (17.4)	135.8 (19.3)	0.515
Diastolic	82.7 (10.2)	79.6 (9.9)	**0.017**
Metabolic components, mmol/l:			
Fasting plasma glucose	6.3 (1.9)	6.3 (1.8)	0.894
Triglycerides	1.7 (1.0)	1.8 (0.9)	0.597
Cholesterol	5.6 (1.2)	5.6 (1.2)	0.949
HDL	1.4 (0.4)	1.4 (0.6)	0.549
LDL	3.6 (1.1)	3.6 (1.0)	0.734
Physical activity level, score:			
ACSM/AHA questionnaire	1.7 (1.5)	1.7 (1.4)	0.975
HRQOL SF-36, score:			
Physical functioning	81.1 (18.3)	70.4 (24.1)	**0.001**
Role limitation, physical	69.9 (37.3)	58.1 (42.1)	**0.030**
Bodily pain	67.0 (26.7)	55.1 (28.1)	**0.001**
General health	60.5 (20.4)	53.5 (20.1)	**0.009**
Vitality	52.4 (23.2)	44.7 (23.6)	**0.010**
Social function	78.9 (25.2)	68.8 (29.2)	**0.007**
Role limitation, emotional	72.9 (39.0)	58.6 (45.2)	**0.015**
Mental health	72.1 (19.9)	65.8 (21.9)	**0.016**
Physical component summary	45.7 (9.9)	41.4 (10.8)	**0.001**
Mental component summary	44.4 (13.1)	40.0 (14.6)	**0.012**

BMI, body mass index; LDL, low density lipoprotein; HDL, high density lipoprotein; ACSM, American College of Sports Medicine; AHA, American Heart Association; HRQOL SF-36, Health Related Quality of Life 36-Item Short Form Health Survey.

^a^ Data are given as mean (standard deviation).

^b^ Difference between follow-up and dropout group. *P*-value for the variables was determined by an independent samples *t*-test.

Statistical significance was set at *p*≤ 0.05.

A higher proportion of women and musculoskeletal disorders and a lower level of SF-36 HRQOL were seen in the dropout group (Tables 1 and 2). There was a significantly lower SF-36 value, not presented in table, for BP (*p* = 0.048) among women compared with men in the dropout group. A lower DBP was also present in the dropout group (Table 2).

In a subgroup analysis between men and women, inferior values were seen in metabolic health (DBP, FPG, TG, Chol, HDL) for men and in HRQOL (PF, BP, SF, RE, MH, PCS) for women at baseline (Table 3).

**Table 3:** Baseline characteristics in anthropometrics, metabolic risk factors, physical activity level and health related quality of life—Men and women.

Variable^a^	Men (n = 193)	Women (n = 251)	*p* value^b^
BMI, kg/m^2^	32.2 (5.0)	32.1 (5.6)	0.788
Waist circumference, cm	113 (12.4)	104 (12.5)	**<0.001**
Blood pressure, mm/Hg:			
Systolic	137.4 (17.1)	136.7 (18.2)	0.668
Diastolic	83.5 (10.9)	81.1 (9.4)	**0.016**
Metabolic components, mmol/l:			
Fasting plasma glucose	6.6 (2.2)	6.0 (1.5)	**0.003**
Triglycerides	2.0 (1.2)	1.5 (0.7)	**<0.001**
Cholesterol	5.4 (1.2)	5.7 (1.2)	**0.004**
HDL	1.2 (0.4)	1.6 (0.5)	**<0.001**
LDL	3.5 (1.0)	3.7 (1.1)	0.168
Physical activity level, score:			
ACSM/AHA questionnaire	1.8 (1.5)	1.7 (1.5)	0.403
HRQOL SF-36, score:			
Physical functioning	83.2 (17.5)	76.3 (20.9)	**<0.001**
Role limitation, physical	71.5 (36.2)	65.1 (39.8)	0.087
Bodily pain	69.2 (26.5)	61.7 (27.4)	**0.005**
General health	59.4 (19.5)	59.2 (21.4)	0.911
Vitality	52.5 (23.2)	50.0 (23.6)	0.275
Social function	80.0 (24.4)	75.0 (27.3)	**0.049**
Role limitation, emotional	76.0 (36.8)	66.2 (42.6)	**0.011**
Mental health	73.2 (19.2)	69.3 (21.0)	**0.049**
Physical component summary	46.2 (9.6)	44.0 (10.5)	**0.035**
Mental component summary	45.0 (12.4)	42.7 (14.2)	0.071

BMI, body mass index; LDL, low density lipoprotein; HDL, high density lipoprotein; ACSM, American College of Sports Medicine; AHA, American Heart Association; HRQOL SF-36, Health Related Quality of Life 36-Item Short Form Health Survey.

^a^ Data are given as mean (standard deviation).

^b^ Difference between men and women. *P*-value for the variables was determined by an independent samples *t*-test.

Statistical significance was set at *p*≤ 0.05.

The three questions regarding the readiness to change PA levels were: How prepared? (mean 81.4 mm, SD 17.7 mm), How important? (mean 83.9 mm, SD 17.9 mm), and How confident? (self-efficacy) (mean 64.3 mm, SD 23.9 mm). A subgroup analyses between the completed group *vs*. dropout group showed significantly lower values for the dropout group regarding confidence to succeed (65.9 mm *v* 56.3 mm, 95% CI: 2.7–16.6, *p* = 0.007).

### Six-month follow-up

Statistically significant positive changes were reached using all four PA instruments (Table 4). The most commonly prescribed PA modality overall was moderate intensity walking, 30–44 min, 2–5 times/week. At the 6-month follow-up, 270 patients (73%, *d-value* = 1.17) in the study group had increased their PA level, measured with the ACSM/AHA questionnaire and a total of 153 patients (42%) had improved their PA from inadequate to sufficient, according to the public health recommendations of the ACSM/AHA. There were also significant improvements in BMI, WC, SBP, FPG, TG, Chol, and LDL with small *d*-values except for women’s WC reaching a medium *d*-value at the 6-month follow-up. The SF-36 showed a significant increase in 6 of 8 health concepts: RP, GH, VT, SF, RE, and MH and in the physical and mental component summary with small *d*-values (Table 5).

**Table 4:** Descriptive statistics and differences for physical activity level at baseline and 6-months follow-up.

Variable (n)	Baseline	6-months follow up	Mean difference (6-months—baseline)	95% CI	*p* value
ACSM/AHA questionnaire, score (361)^a^	1.75 (1.55)	4.57 (3.29)	2.8 (3.4)	2.5;3.2	**<0.001**^c^
IPAQ 1–3, score (236)^b^	1 (1–2)	2 (1–2)	-	-	**<0.001**^d^
IPAQ 1–3, category, No (%)					
• Low	222 (62.4)	130 (47.3)	-	-	
• Moderate	134 (37.6)	145 (52.7)	-	-	
• High	0	0	-	-	
SGPALS 1–4, score (337)^b^	2 (1–3)	2 (1–3)	-	-	**<0.001**^d^
SGPALS 1–4, category, No (%)					
• 1	158 (36.5)	66 (19.2)	-	-	
• 2	268 (61.9)	223 (65.1)	-	-	
• 3	7 (1.6)	54 (15.7)	-	-	
• 4	0	0	-	-	
Frändin/Grimby 1–6, score (338)^b^	3 (1–5)	3 (1–6)	-	-	**<0.001**^d^
Frändin/Grimby 1–6, category, No (%)					
• 1	31 (7.1)	7 (2.0)	-	-	
• 2	75 (17.3)	41 (12.0)	-	-	
• 3	243 (56.0)	169 (49.1)	-	-	
• 4	81 (18.7)	107 (31.1)	-	-	
• 5	4 (0.9)	17 (4.9)	-	-	
• 6	0	3 (0.9)	-	-	

CI, confidence intervals; ACSM, American College of Sports Medicine; AHA, American Heart Association; IPAQ; International Physical Activity Questionnaire

^a^Values are given as mean (standard deviation).

^b^Values are given as median (minimum-maximum).

^c^*P* values were determined by a paired samples *t*-test for the difference between baseline and 6-months follow up.

^d^*P* values were determined by Wilcoxon Signed Ranks Test for the difference between baseline and 6-months follow up.

Statistical significance was set at *p*≤ 0.05.

**Table 5:** Descriptive statistics and differences for anthropometric-, metabolic characteristics and health related quality of life at baseline and 6-month follow-up.

Variable^a^ (n)	Baseline	6-months follow up	Mean difference (6-month—baseline)	95% CI	*p* value^b^	Cohen’s *d*^c^
BMI, kg/m^2^ (353)	32.0 (5.2)	31.7 (5.4)	-0.3 (1.7)	-0.5;-0.1	**0.001**	0.25
Waist circumference, cm (352)	107.8 (13.2)	106.2 (13.9)	-1.7 (5.8)	-2.3;-1.1	**<0.001**	0.41
• female (187)	103.4 (12.2)	101.4 (13.2)	-2.1 (5.9)	-2.9;-1.2	**<0.001**	0.50
• male (165)	112.8 (12.6)	111.6 (12.6)	-1.3 (5.6)	-2.1;-0.4	**0.005**	0.32
Blood pressure, mm/Hg:						
Systolic (358)	137.5 (17.3)	133.9 (16.2)	-3.6 (16.4)	-5.3;-1.9	**<0.001**	0.31
Diastolic (358)	82.8 (10.1)	82.5 (9.3)	-0.4 (9.9)	-1.4;0.6	0.466	0.05
Metabolic components, mmol/l:						
Fasting plasma glucose (352)	6.26 (1.92)	6.01 (1.44)	-0.3 (1.2)	-0.4;-0.1	**<0.001**	0.29
Triglycerides (355)	1.69 (0.99)	1.59 (0.88)	-0.1 (0.8)	-0.2;0.0	**0.016**	0.18
Cholesterol (358)	5.57 (1.21)	5.39 (1.16)	-0.2 (0.9)	-0.3;-0.1	**<0.001**	0.27
HDL (357)	1.41 (0.45)	1.43 (0.45)	0.0 (0.3)	0.0;0.1	0.196	0.10
LDL (353)	3.63 (1.06)	3.52 (1.03)	-0.1 (0.8)	-0.2;0.0	**0.009**	0.20
HRQOL SF-36, score:						
Physical functioning (335)	81.3 (18.1)	81.8 (19.1)	0.4 (14.1)	-1.1;2.0	0.558	0.04
Role limitation, physical (323)	70.0 (37.4)	77.4 (33.3)	7.4 (39.7)	3.1;11,8	**0.001**	0.26
Bodily pain (334)	67.3 (26.6)	69.6 (27.2)	2.3 (22.9)	-0.2;4.7	0.069	0.14
General health (335)	60.7 (20.2)	64.2 (20.8)	3.6 (14.4)	2.0;5.1	**<0.001**	0.35
Vitality (333)	52.6 (23.1)	58.3 (21.6)	5.7 (19.4)	3.6;7.8	**<0.001**	0.42
Social function (334)	79.2 (25.2)	83.6 (21.8)	4.4 (24.6)	1.8;7.1	**0.001**	0.26
Role limitation, emotional (324)	73.4 (38.8)	77.8 (36.2)	4.4 (39.0)	0.2;8.7	**0.042**	0.16
Mental health (333)	72.2 (19.8)	74.3 (18.8)	2.2 (16.3)	0.4;3.9	**0.017**^b^	0.19
Physical component summary (318)	45.8 (9.9)	46.8 (9.9)	1.0 (8.0)	0.1;1.9	**0.029**	0.17
Mental component summary (318)	44.6 (13.2)	46.6 (11.8)	2.0 (10.9)	0.8;3.2	**0.001**	0.19

CI, confidence intervals; BMI, body mass index; HDL, high density lipoprotein; LDL, low density lipoprotein; HRQOL SF-36, Health Related Quality of Life 36-Item Short Form Health Survey.

^a^Values are given as mean (standard deviation).

^b^*P* values were determined by a paired samples *t*-test for the difference between baseline and 6-months follow up.

^c^Effect size in within-subjects comparisons ($\text{Cohen′s }d_{\text{z}}\;\times \sqrt{2}\;=\text{Cohen′s }d$) was measured quantifying the degree of differentiation in values between baseline and 6-months follow-up.

Statistical significance was set at *p*≤ 0.05.

A multivariate regression analysis showed positive significant associations between changes in the PA level and the health outcomes of metabolic risk factors (Pillai’s Trace = 0.063, *p* = 0.032) and SF-36 HRQOL (Pillai’s Trace = 0.095, *p*<0.001) at the 6-month follow-up. Univariate linear regression analysis showed positive significant associations between changes in the PA level and the BMI (Table 6), and the SF-36: RP, BP, GH, VT, SF, MH, PCS, and MCS (Table 7). The univariate regression models could only partially explain how the changes in the PA level were related to metabolic risk factor outcomes (R^2^-values 0.03–0.05) and health concepts in the SF-36 (R^2^-values 0.03–0.12).

**Table 6:** Results for univariate linear regression analysis investigating the association between change in PA and metabolic risk factors at 6-month follow-up^a^.

Dependent variable	Independent variable	β	95% CI	*p* value
Δ BMI, kg/m^2^	Δ Change PA	-0.069	-0.128;-0.010	**0.022**
Δ Waist circumference, cm	Δ Change PA	-0.124	-0.340;0.091	0.256
Δ Blood pressure systolic, mm/Hg	Δ Change PA	0.489	-0.108;1.086	0.108
Δ Blood pressure diastolic, mm/Hg	Δ Change PA	0.277	-0.083;0.637	0.131
Δ Fasting plasma glucose, mmol/l	Δ Change PA	-0.035	-0.076;0.006	0.097
Δ Triglycerides, mmol/l	Δ Change PA	-0.013	-0.039;0.014	0.341
Δ Cholesterol, mmol/l	Δ Change PA	-0.025	-0.059;0.008	0.135
Δ HDL, mmol/l	Δ Change PA	0.001	-0.007;0.009	0.739
Δ LDL, mmol/l	Δ Change PA	-0.012	-0.042;0.017	0.410

PA, physical activity according to ACSM/AHA questionnaire; β, change in value; CI, confidence intervals; Δ, the difference between 6-month value and start value; BMI, body mass index; HDL, high density lipoprotein; LDL, low density lipoprotein.

^a^Adjusted for PA level at baseline, age, sex, social situation, economy, education and smoking.

Statistical significance was set at *p*≤ 0.05.

**Table 7:** Results for univariate linear regression analysis investigating the association between change in PA and health related quality of life (SF-36) at 6-month follow-up^a^.

Dependent variable	Independent variable	β	95% CI	*p* value
HRQOL SF-36, score:				
Δ Physical functioning	Δ Change PA	0.432	-0.077;0.941	0.096
Δ Role limitation, physical	Δ Change PA	1.713	0.289;3.138	**0.019**
Δ Bodily pain	Δ Change PA	1.184	0.380;1.988	**0.004**
Δ General health	Δ Change PA	0.785	0.265;1.306	**0.003**
Δ Vitality	Δ Change PA	1.702	1.017;2.388	**<0.001**
Δ Social function	Δ Change PA	1.435	-0.561;2.309	**0.001**
Δ Role limitation, emotional	Δ Change PA	1.004	-0.449;2.456	0.175
Δ Mental health	Δ Change PA	1.171	0.576;1.765	**<0.001**
Δ Physical component summary	Δ Change PA	0.338	0.048;0.628	**0.022**
Δ Mental component summary	Δ Change PA	0.659	0.265;1.053	**0.001**

PA, physical activity according to ACSM/AHA questionnaire; β, change in value; CI, confidence intervals; HRQOL SF-36, Health Related Quality of Life 36-Item Short Form Health Survey; Δ, the difference between 6-month value and start value.

^a^Adjusted for PA level at baseline, age, sex, social situation, economy, education and smoking.

Statistical significance was set at *p*≤ 0.05.

In a within subgroup analysis between men and women, there were improved metabolic risk factor values for both men (6 of 9 measured parameters) and women (5 of 9) at the 6-month follow-up. Increased HRQOL values were seen in 2 of 10 health concepts among men and 7 of 10 among women (Table 8). In a between group analysis, not presented in table, women increased their PA level more than men.

**Table 8:** Descriptive statistics and differences for anthropometric-, metabolic characteristics, physical activity level and health related quality of life at baseline and 6-month follow-up—Men and women.

Variable^a^ (n; men/women)	Men			Women		
	Mean diff 6-months—baseline	95% CI	*p* value^b^	Mean diff 6-months—baseline	95% CI	*p* value^b^
BMI, kg/m^2^ (164/189)	-0.18	-0.43;0.06	0.139	-0.39	-0.64;-0.14	**0.002**
Waist circumference, cm (165/187)	-1.26	-2.12;-0.39	**0.005**	-2.09	-2.94;-1.23	**<0.001**
Blood pressure, mm/Hg:						
Systolic (166/192)	-3.38	-5.74;-1.01	**0.005**	-3.76	-6.21;-1.29	**0.003**
Diastolic (166/192)	-0.04	-1.59;1.51	0.957	-0.68	-2.08;0.72	0.342
Metabolic components, mmol/l:						
Fasting plasma glucose (161/191)	-0.36	-0.56;-0.14	**0.001**	-0.17	-0.33;-0.01	**0.032**
Triglycerides (163/192)	-0.18	-0.33;0.02	**0.020**	-0.03	-0.10;0.04	0.417
Cholesterol (163/195)	-0.23	-0.37;-0.07	**0.003**	-0.14	-0.27;-0.01	**0.039**
HDL (164/193)	0.02	-0.03;0.07	0.464	0.02	-0.01;0.05	0.213
LDL (161/192)	-0.17	-0.30;-0.03	**0.017**	-0.08	-0.20;0.04	0.184
Physical activity level, score:						
ACSM/AHA questionnaire (168/193)	2.44	1.97;2.91	**<0.001**	3.16	2.63;3.68	**<0.001**
HRQOL SF-36, score:						
Physical functioning (153/182)	-1.2	-3.53;1.05	0.286	1.9	-0.14;3.89	0.068
Role limitation, physical (148/175)	6.2	-0.02;12.41	0.051	8.5	2.4;14.64	**0.007**
Bodily pain (152/182)	2.8	-0.71;6.31	0.117	1.8	-1.62;5.32	0.294
General health (153/182)	3.2	0.98;5.51	**0.005**	3.8	1.72;5.99	**<0.001**
Vitality (152/181)	6.2	3.70;8.71	**<0.001**	5.3	2.10;8.58	**0.001**
Social function (153/181)	2.5	-1.11;6.18	0.172	6.1	2.27;9.88	**0.002**
Role limitation, emotional (146/178)	0.2	-5.62;6.08	0.939	7.9	1.76;13.97	**0.012**
Mental health (152/181)	0.2	-2.32;2.67	0.888	3.8	1.35;6.28	**0.003**
Physical component summary (144/174)	1.0	-0.29;2.27	0.128	1.0	-0.25;2.22	0.117
Mental component summary (144/174)	0.9	-0.81;2.53	0.311	3.0	1.28;4.72	**0.001**

CI, confidence intervals; BMI, body mass index; HDL, high density lipoprotein; LDL, low density lipoprotein; ACSM, American College of Sports Medicine; AHA, American Heart Association; IPAQ; International Physical Activity Questionnaire; HRQOL SF-36, Health Related Quality of Life 36-Item Short Form Health Survey.

^a^Values are given as mean.

^b^*P* values were determined by a paired samples *t*-test for the difference between baseline and 6-months follow up.

Statistical significance was set at *p*≤ 0.05.

**Fig 2:**
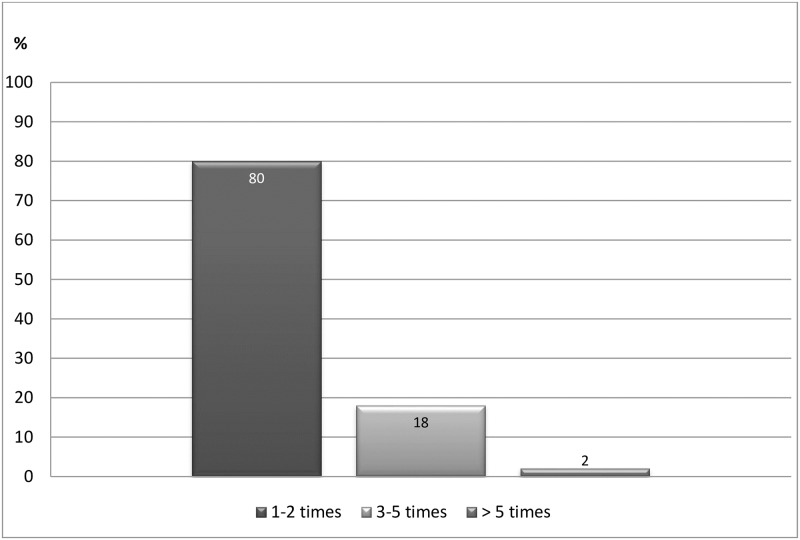
The relative distribution of support from the PAP-responsible nurse at the health care centre. Was measured at the 6 months follow-up with a question, to the patient, about contact frequency.

During the first 6 month period, excluding the start visit, 80% of the patients received PAP support from caregivers 1–2 times, either through visits at the health care center or by telephone contact (Fig 2).

## Discussion

The main results of this study were the improvements in PA level, metabolic risk factors, and self-reported quality of life at the 6 month follow-up of PAP-treatment in primary health care for 27–85 year olds, having at least one component of MetS. Multivariate regression analysis also showed positive significant associations between changes in the PA level and health outcomes, similar to previous studies [44, 45].

To explore the association between PAP-treatment and the PA level, four self-reported questionnaires were used and all showed significant increments in the PA level. The difference measured with the ACSM/AHA questionnaire had a *d* value (*d* = 1.17) that indicated a large effect size. The finding that several PA instruments showed positive changes increases the dependability of the overall result and may be important since the concept of PA is contextualized, complex and difficult to measure [46]. Two previous meta-analyses [11, 12] regarding the clinical effects of PAP-interventions also demonstrated difficulties with measuring PA due to heterogeneity in the quality and nature of the studies. In addition, several of the included studies had a fixed short-time intervention (10–12 weeks) [47–50] and were linked to predetermined activities, e.g. leisure center-based or community walk programs [48–51], and not individualized to the needs and opinions of the patients. There was also uncertainty about the customized structured support and follow-up during the intervention. The lack of patient-related individualization of the PAP-intervention may have influenced the outcome. This present study was a totally individualized intervention and the PAP-treatment consisted of individual-based dialogue with the patient, an individually tailored recommendation of PA, and customized, structured support over 6 months. The majority of the patients chose a PA to be carried out, on their own, in everyday life near their residential area or workplace. The majority of the patients received PAP support 1–2 times during the 6 month period; thus, the effort made by the primary health care workers was small and the cost was relatively low [52].

The present study showed significant improvements in the majority of metabolic risk factors, measured at the 6-month follow-up and positive associations between changes in the PA level and metabolic health outcomes. Previous PAP studies have shown some, but varied outcomes regarding metabolic risk factors [47, 49, 50] in within group analyses, but often non-significant effects in the between group analysis. Though, Kallings et al. [15], in a Swedish PAP study among elderly subjects, showed an increase in metabolic health in both within- and between group analyses with patient-individualized PAP-intervention versus usual care. In this study, the results were even more obvious with regard to the effects on metabolic risk factors and may strengthen the individualization perspective of the PAP-treatment. The relatively small effect sizes regarding changes in metabolic parameters can be explained by the existing dose-response relationship between PA level and health outcomes [44, 45]. In this study, 42% of the patients reached the public health recommendations and the most commonly prescribed exertion was moderate intensity. A more extensive increase in PA level among the patients not reaching the public health recommendations or PA on a vigorous-intensity level possibly would have increased the effect size.

Even HRQOL, measured with the SF-36, was significantly improved for 8 of 10 health concepts with a small effect size (*d*) and five of the concepts considered clinically relevant [53]. Small differences on the SF-36 health survey (i.e. 3- to 5-points) are considered clinically important even if the effect size is estimated as small [54, 55]. Importantly, the improvements in our study were associated with changes in the PA level. Several previous PAP studies found improvements in quality of life [17, 47, 49, 51, 56–58]. However, the studies that used the SF-36 reported smaller numbers of improved health concepts, with the exception of two Swedish studies showing significant positive changes in the majority of health concepts at 6 months [59] and 2 years follow-up [60]. This study found similar changes in HRQOL, although the inclusion criteria were different. However, the amount of PAP support is only reported in the present study, where 80% of the patients received support from caregivers 1–2 times during the 6 month period.

Although univariate regression models showed positive significant associations, they could not explain all the changes in health outcomes. An increased PA level explained 3–5% of the metabolic health effects and 3–12% of the health effects in the HRQOL SF-36. However, the role of PA is difficult and complex to measure and not fully clarified due to its multifactorial effects. The relatively low correlation between changes in physical fitness and metabolic risk factors has been discussed due to exercise-induced improvements in body composition, e.g. reduced abdominal visceral fat, per se, has positive effects on the metabolic risk profile [61].

There were some differences in baseline characteristics between those who could be followed up at 6 months and the dropout group. The most interesting findings were that women with an additional musculoskeletal diagnosis and lower self-reported quality of life (BP *p* = 0.048), were more frequent in the dropout group. This might give an indication that some patients are in need of more enhanced support to increase their PA level or would benefit from a period of rehabilitation before or in combination with a PAP intervention.

There were some differences between men and women, within the study group, with inferior values in metabolic variables for men and HRQOL for women at baseline. At the 6 month follow-up, the women had increased their PA and HRQOL values more than men; whereas, the improvements in metabolic parameters were similar between the groups. In contrast, previous studies of lifestyle interventions have shown that men were more likely to increase their PA level [62, 63]. The most common recommendation for PAP was for PA to be carried out in everyday life (e.g. taking walks) near the subject’s residential area or workplace, a recommendation which may be suitable for many women. These gender-related differences should be considered when individualizing the intervention and support for the patient. Various subgroup analyses are needed to explore the possible needs for the patient to succeed when making this behavioral change.

### Limitations

The dropout rate was 17% between baseline and the 6-month follow-up, and this may be normal in this type of study [59, 64]. Importantly, the present study was a “daily clinical work” survey with ordinary primary health care patients visiting their local health care center where the personnel had no extra time to manage this part of their duties. A per-protocol analysis was used where the drop-out group was excluded from the analysis between the baseline and 6-month follow-up, a method that increases the risk of bias. Although, when using an intention to treat analysis (ITT) that includes the dropout group, there would be a risk of attributing characteristics to the patients in the dropout group that they did not have. The ITT analysis has been criticized for potentially leading to a biased treatment effect [65, 66].

This survey was an observational, follow-up study in daily clinical practice without any control group. The lack of a control group complicates the interpretation of estimating the effects of the PAP-intervention on the increased PA level and positive health effects. However, the results in this study are comparable to other studies evaluating intervention groups in randomized controlled trials (RCTs) [15, 64] and two Swedish PAP studies without a control group that reported increased PA levels [16, 59]. Notably, a review assessing the impact of RCTs versus an observational study design revealed no differences in effect estimates (pooled ratio of odds ratios of 1.08) [67]. In addition, there are limitations of RCTs due to the possible lack of external validity resulting in non-appropriate evidence on which clinical decisions are based [68, 69].

An observational study design requires caution concerning bias that can undermine the internal validity [70]. The study population was non-consecutively included. No data were collected for patients with the same inclusion criteria who visited the health care centers and were not included in the study. It is not possible to estimate how many patients, in total, were candidates for treatment with PAP. The risk of selection bias increases when including patients more willing to change their PA level. The patients estimated high values for how prepared they were and how important it was to increase their PA level, but lower values for the confidence (self-efficacy) to succeed. The dropout group had even lower values for self-efficacy (*p* = 0.007), which is predictive for exercise adherence and compliance in both the adoption and maintenance of PA among adults [71, 72]. The PAP-treatment method is patient-centered and dependent on the patient’s attitude to change living habits and the health care professional’s ability to evaluate what stage of change the patient is in. The PAP method probably has the most potential for patients in contemplation or preparation stages [73].

Using multiple measurements to assess the outcomes may incur a risk for type I errors. However, multiple predefined outcomes in this study were needed to indicate several effects and they seem to be justified without the need for adjustment [74]. Measuring PA seems to be a complex issue with methodological problems due to the diversified designs of several studies [75]. Using self-reported questionnaires may increase the risk of recall or social desirability bias, but is both practical and valid [64]. In this study, we used four self-reported instruments, and all showed similar results.

## Conclusions

This observational study indicated that an individual-based PAP-treatment has the potential to change people’s PA behavior with improved metabolic risk factors and self-reported quality of life at the 6-month follow-up. The PAP-treatment proved to work in daily clinical primary care with educated, authorized personnel, structured routines, and readily available information for both patients and workers. If implemented widely, PAP-treatment has the potential to become an important method and may result in major health benefits for physically inactive, sedentary patients with a minimum effort from healthcare professionals. However, further research about the outcome of PAP-treatment strategies is needed regarding individualized, well-defined interventions and long term follow-up in RCTs.

## Supporting information

S1 File Source data file. (XLSX)
